# Effects of environmental Bisphenol A exposures on germ cell development and Leydig cell function in the human fetal testis

**DOI:** 10.1371/journal.pone.0191934

**Published:** 2018-01-31

**Authors:** Soria Eladak, Delphine Moison, Marie-Justine Guerquin, Gabriele Matilionyte, Karen Kilcoyne, Thierry N’Tumba-Byn, Sébastien Messiaen, Yoann Deceuninck, Stéphanie Pozzi-Gaudin, Alexandra Benachi, Gabriel Livera, Jean-Philippe Antignac, Rod Mitchell, Virginie Rouiller-Fabre, René Habert

**Affiliations:** 1 Univ. Paris Diderot, Sorbonne Paris Cité, Laboratory of Development of the Gonads, Unit of Genetic Stability, Stem Cells and Radiation, Fontenay-aux-Roses, France; 2 CEA, DSV, iRCM, SCSR, LDG, Fontenay-aux-Roses, France; 3 INSERM, Unité 967, Fontenay aux Roses, France; 4 MRC Centre for Reproductive Health, The University of Edinburgh, The Queen's Medical Research Institute, Edinburgh, Scotland, United Kingdom; 5 Laboratoire d’Etude des Résidus et Contaminants dans les Aliments (LABERCA), Ecole Nationale Vétérinaire Agroalimentaire et de l’Alimentation Nantes Atlantique (ONIRIS), Nantes, France; 6 Service de Gynécologie-Obstétrique et Médecine de la Reproduction, Hôpital A. Béclère, Université Paris Sud, Clamart, France; Universite Clermont Auvergne, FRANCE

## Abstract

**Background:**

Using an organotypic culture system termed human Fetal Testis Assay (hFeTA) we previously showed that 0.01 μM BPA decreases basal, but not LH-stimulated, testosterone secreted by the first trimester human fetal testis. The present study was conducted to determine the potential for a long-term antiandrogenic effect of BPA using a xenograft model, and also to study the effect of BPA on germ cell development using both the hFETA and xenograft models.

**Methods:**

Using the hFeTA system, first trimester testes were cultured for 3 days with 0.01 to 10 μM BPA. For xenografts, adult castrate male nude mice were injected with hCG and grafted with first trimester testes. Host mice received 10 μM BPA (~ 500 μg/kg/day) in their drinking water for 5 weeks. Plasma levels of total and unconjugated BPA were 0.10 μM and 0.038 μM respectively. Mice grafted with second trimester testes received 0.5 and 50 μg/kg/day BPA by oral gavage for 5 weeks.

**Results:**

With first trimester human testes, using the hFeTA model, 10 μM BPA increased germ cell apoptosis. In xenografts, germ cell density was also reduced by BPA exposure. Importantly, BPA exposure significantly decreased the percentage of germ cells expressing the pluripotency marker AP-2γ, whilst the percentage of those expressing the pre-spermatogonial marker MAGE-A4 significantly increased. BPA exposure did not affect hCG-stimulated androgen production in first and second trimester xenografts as evaluated by both plasma testosterone level and seminal vesicle weight in host mice.

**Conclusions:**

Exposure to BPA at environmentally relevant concentrations impairs germ cell development in first trimester human fetal testis, whilst gonadotrophin-stimulated testosterone production was unaffected in both first and second trimester testis. Studies using first trimester human fetal testis demonstrate the complementarity of the FeTA and xenograft models for determining the respective short-term and long term effects of environmental exposures.

## Introduction

Over recent decades, the incidence of male reproductive disorders has been steadily increasing [1–4]. These disorders such as cryptorchidism, hypospadias, low sperm count and quality, and testicular cancer are hypothesized to arise from abnormal development of the fetal testis. These associated disorders have been collectively described as a testicular dysgenesis syndrome (TDS) [5–8].

In 1993, Sharpe and Skakkebaek hypothesized that endocrine disruptors (EDs), particularly EDs with an estrogenic effect, could be an explanation for the increase in male reproductive disorders [9] initiating a large number of studies in reproductive toxicology [4,10,11]. Among such EDs, bisphenol A (BPA; 4,4'-dihydroxy-2,2-diphenylpropane) has been the focus of considerable research [12–15].

BPA is one of the most frequently produced synthetic chemicals worldwide, with approximately 70% used to produce polycarbonate plastics for a variety of products, including housewares and appliances, opticals, construction materials and medical, packaging. A further 20% of BPA is used as an essential component of epoxy resins that are mainly used to coat the inner surface of metallic food and beverage cans. Finally, BPA is used as antioxidant or inhibitor of polymerization in some plasticizers, polyvinyl chloride, and thermal cash register paper.

Many studies have shown that BPA exposure of rodents during intrauterine life can induce a range of adverse effects in adult testes. It has been shown that *in utero* or perinatal BPA exposure induces a decrease in sperm quality and production and testosterone secretion in adults [14,16–21]. These results suggest that BPA potentially disturbs fetal testis development and future function.

However, there is limited data and conflicting results concerning the direct immediate effect of *in vivo* BPA exposure on fetal testis development and function. In pregnant rats, exposure to high doses of BPA (876 μM *i*.*e*. calculated to be approximately 20,000 μg/kg/day, based on an adult rat weight of 300 g and a fluid intake of about 30 mL/day) in drinking water reduced plasma testosterone in the offspring at birth [22]. Gavage of pregnant rats with 1–200 mg/kg/day BPA increased the mRNA level but not the protein levels of Raf1 in the Leydig cells at postnatal day 3 [23]. Administration of 250 μg/kg/day BPA reduced the anogenital distance in male rat pups, which suggests a reduction in fetal androgen exposure, whereas lower BPA doses did not have any effect [24]. On the contrary, three other studies investigating potential effects of BPA exposure, found no difference in AGD between BPA and vehicle exposed pups following gestational gavage with doses equal or higher than 1 mg/kg/day and reaching 200 to 50000 mg/kg/day [25–27].

Furthermore, recent *ex vivo* analyses have demonstrated the complexity of the potential effect of BPA on Leydig cell function and development. Using an organotypic culture system termed “Fetal Testis Assay” (FeTA) developed for rat fetal testis in 1990's [28] and extended for mouse and human fetal testes thereafter [29,30], we demonstrated that BPA concentrations as low as 0.01 μM (*i*.*e*. 2.28 μg/L) reduced basal testosterone secretion and Insl-3 expression by the human fetal testis, but not by mouse and rat fetal testes for which concentrations equal or higher to 1 μM BPA were required in order to induce similar effects [31]. This was confirmed for human fetal testes subsequently by another laboratory using the same FeTA approach [32]. In the continuous presence of LH or hCG, 1 or 10 μM BPA was necessary to reduce testosterone secretion in human, mouse and rat [15,32].

To date, no study has investigated the effect of BPA on male germ cell (GC) development during fetal life in either rodent or human models. Fetal primordial GC migrate into the embryonic genital ridge. By 7 weeks of gestation in human they are enclosed in the seminiferous cords at which point they are termed gonocytes [33–36]. Gonocytes express the pluripotency markers such as Activating Enhancer Binding Protein 2 Gamma (AP-2y), a transcription factor associated with an undifferentiated state and encoded by the *TFAP2C* gene. As GC differentiate from gonocytes to pre-spermatogonia the expression of AP-2y is reduced and the cells begin to express Melanoma Associated Antigen A4 (MAGE-A4) a differentiation marker also expressed in adult spermatogonia [37–42]. In rodents, this transition from gonocyte to pre-spermatogonia occurs synchronously during late gestation, whereas in humans this transition occurs asynchronously over the remainder of fetal and into early postnatal life [37,38,40,42–44]. During the first trimester of gestation, we have previously shown that the rate of GC proliferation is ~25–30%, and ~1–3% of GC are apoptotic [36]. In the second trimester ~20–25% of gonocytes are proliferative, compared with only ~3% of pre-spermatogonia at this stage [41].

The present study was designed to investigate the effects of BPA on both endocrine function and GC development in the human fetal testis. We combined two experimental models: we used the ‘short term’ organotypic culture system (FeTA) and the ‘long term’ xenograft system [41,45,46], both of which have been validated for assessment of effects of exposures to chemicals on human fetal testis development.

The specific aims were 1) to investigate the potential for an antiandrogenic effects of BPA in human fetal testes from the first and the second trimesters using the xenograft system 2) to study the effect of BPA on GC development in first trimester testes using both FeTA and xenograft systems, 3) to compare the results of the two experimental models using first trimester testis tissue.

## Materials and methods

### First trimester human Fetal Testis Assay (hFeTA) and xenograft studies

#### Collection of human fetal testis

Human fetal testes (5–12 Gestational Weeks; GW) were obtained from pregnant women referred to the Department of Gynecology and Obstetrics at the Antoine Béclère hospital (Clamart, France) for legally induced abortions in the first trimester of pregnancy as previously described [30]. All women provided written informed consent for scientific use of the fetal tissues. None of the abortions were due to fetal abnormality. The fetal age was determined by measuring the length of limbs and feet [47]. Gonads within the abortive material were retrieved in 50% of cases. Testes were identified based on their size and their morphology. The project was approved by the local Medical Ethics Committee and by the French Biomedicine Agency (reference number PFS 12–002).

#### Human Fetal Testis Assay (hFeTA)

Human Fetal Testis Assay (hFeTA) is the organotypic culture system developed in our laboratory and largely described previously [28–30,48,49]. Briefly, human testes were cut into small pieces (12 to 30 pieces depending on the age of the fetus) and 3–4 pieces were randomly placed on Millicell-CM Biopore membranes (pore size 0.4 μm, Millipore, Billerica, MA) floating on 320 μL culture medium in tissue culture dishes. Cultures were performed at 37°C in a humidified atmosphere containing 95% air/ 5% CO_2_. The culture medium was phenol-red free Dulbecco modified Eagle medium/Ham F12 (1:1; Gibco, Grand Island, NY), supplemented with 80 μg/mL gentamicin (Sigma, St. Louis, MO). BPA was purchased from Sigma (CAS no. 80-09-1; Sigma, St. Louis, MO) and diluted in ethanol.

After 24 hours in control medium, explants were cultured with 100 ng/mL LH from human pituitary (≥ 5,000 IU/mg; Sigma) in the presence of ethanol vehicle (control explants) or BPA at concentrations ranging from 0.01 to 10 μM for three subsequent days. Control and treated explants were from the same testis. The whole culture medium was changed every 24h. At the end of the culture, explants were fixed in Bouin’s and formaldehyde for analysis of cell apoptosis and proliferation of germ cells as previously described [30,48].

#### Host mice for xenografting

Nude mice (NMRI-Foxnl^nu^/foxnl^nu^) from Janvier (Saint Berthevin, France) were received at 4 weeks-old and housed in our animal facility under controlled photoperiod conditions (lights on 08:00 am to 08:00 pm). They were supplied with estrogen-free commercial food and tap water ad libitum. Mice received water via glass water bottles. The animal facility is licensed by the French Ministry of Agriculture (agreement n° B92-032-02). All efforts were made to avoid or minimize animal suffering.

#### Xenografting procedure

Pieces of human fetal testis were xenografted into the muscle of the back as previously described for ovarian xenografts [50]. Briefly, both human fetal testes from 9 fetuses (9.1 to 11.3 GW) were cut into pieces (~1 mm^3^ approx.). Male Nude mice (aged 5–8 weeks) were anesthetized by isoflurane inhalation. All pieces from one testis were grafted in one mouse which was then exposed to BPA whilst all the pieces from the other testis of the same human fetus were grafted into another mouse which will serve as a control. The testicular pieces were inserted in the left back muscle of the host mice. Mice were then immediately castrated and injected subcutaneously with 20 IU hCG (Human Chorionic Gonadotrophin, MSD France). Mice received analgesia (Carprofen, 25μg/mL, Santa Cruz, sc 205621) in the drinking water for one recovery week post-surgery.

**Fig 1:**
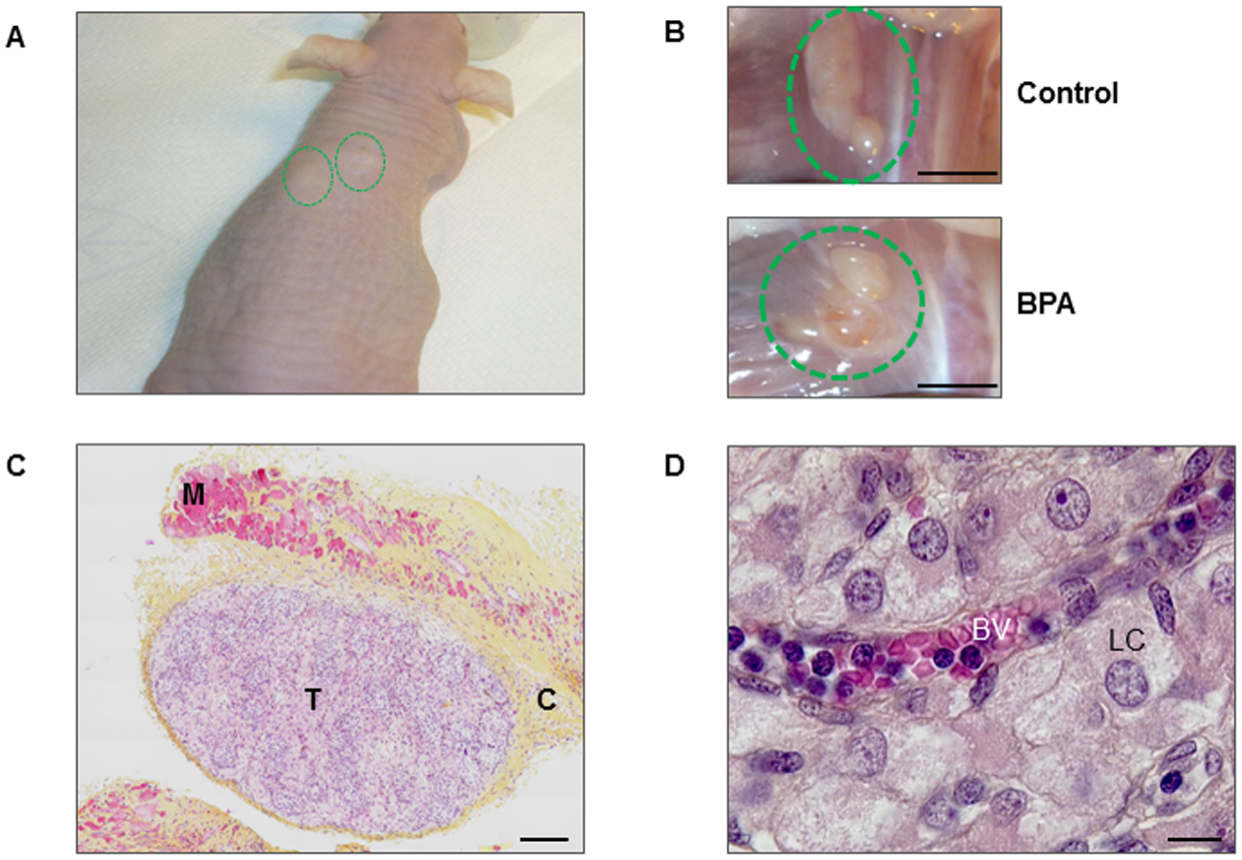
Xenografting first trimester human fetal testis. Human fetal testes (9.1–11.3 GW) xenografted in castrated Nude (host) mice. Host mice received vehicle (Control) or 10μM BPA in the drinking water. (A) Host mouse six weeks after xenografting demonstrating xenograft tissue below the skin (green circles). (B) Xenografted explants (green circles) in the back muscle of the host mouse at the end of the experiment, in a control and a BPA-treated mouse. Scale bar: 5 mm. (C) Histological section of a xenograft after haematoxylin-eosin-saffron staining, T: testis; M: mouse muscle; C: connective tissue. Scale bar: 100 μm. (D) Histological section of a xenograft showing a blood vessel (BV) in the interstitial tissue. LC: Leydig cell. Scale bar: 10 μm.

Six weeks post-xenografting, the xenografted testes were visible as subcutaneous swellings of the host mouse indicating growth of the xenografts in this *in vivo* model (Fig 1A). Xenografts were approximately five times larger than they were when grafted. After euthanasia the xenografts were visible in the back muscle by their white color and their large size in both control and BPA-treated host mice, (Fig 1B).

Histological sections of both control and BPA-treated xenografted testes exhibited a well-maintained architecture with Sertoli cells surrounding the germ cells in the seminiferous cords and the Leydig cells in the interstitial compartment (Fig 1C and 1D). Furthermore, in histological sections blood vessels were present inside the xenografted tissues. There was no evidence of necrosis, demonstrating that the size of xenografted pieces of testis was suitable for supporting good vascularization of tissues and that the xenografting procedure preserved normal histological appearance of the testis (Fig 1C and 1D).

#### Treatment of host mice

All the mice were injected subcutaneously every 72h with 20 IU hCG from the day of surgery until euthanasia. After one recovery week, host mice received drinking water containing 0.1% ethanol (control mice) or BPA diluted in 0.1% ethanol to obtain 10 μM BPA as the final concentration (BPA-treated mice) for the five subsequent weeks. During the treatment period, mice were weighed every week. BPA treatment did not affect the body weight of the mice compared to controls. As one adult mouse weighs ~30 g and drinks ~7 mL of water per day the evaluated daily intake of BPA by treated mice is ~500 μg/kg of body weight/day. For comparison, the US Food and Drug Administration (FDA) and the European Food Safety Authority (EFSA) have determined the NOAEL for BPA to be equal to 5,000 and 8,960 μg/kg of body weight/day in rodents respectively.

#### Sampling in xenograft model

At the end of the grafting period, host mice were anesthetized by isoflurane inhalation and blood was extracted by cardiac puncture for testosterone and BPA measurements. Mice were killed by cervical dislocation and seminal vesicles were removed and weighed. Xenografts were retrieved and fixed in Bouin’s fluid overnight.

#### Internal BPA concentration in the host mice

In order to characterize this model, free and total (free + glucuronide and sulfate conjugates) BPA concentration levels were assayed in plasma of both exposed and control mice. Each sample (20 to 50 μL) was fortified with 10 ng of ^13^C-BPA (internal standard for quantification according to the isotope dilution method), vortexed for 30 sec and followed by equilibration for 3h. For a total BPA determination, i.e.: free + phase II metabolites (glucuronide + sulfate), an enzymatic hydrolysis was carried out using a purified *Helix pomatia* preparation (50°C for 12h). Ethyl acetate (500 μL) was added, the sample was shaken for around 30 sec and centrifuged at 5000 rpm for 15 min at 4°C. The organic phase was collected into a 4 mL glass tube. This liquid/liquid extraction was repeated twice. The resulting organic phase was evaporated to dryness under a gentle nitrogen stream (45°C). The dry residue was reconstituted in 100 μL acetonitrile and transferred to an injection vial, and finally evaporated to dryness. Then, 20 μL of MSTFA [N-Methyl-N-(trimethylsilyl) trifluoroacetamide] were added to the dry residue and the vial was heated for 30 min at 45°C. Detection was achieved using gas chromatography coupled to tandem mass spectrometry (GC-MS/MS) operating in selected reaction monitoring (SRM) acquisition mode as previously described [51,52]. The limit of detection of BPA in the plasma was 0.13 nM (0.03μg/L) and the limit of quantitation was 0.44 nM (0.1μg/L). All the samples were above the limit of quantitation.

**Fig 2:**
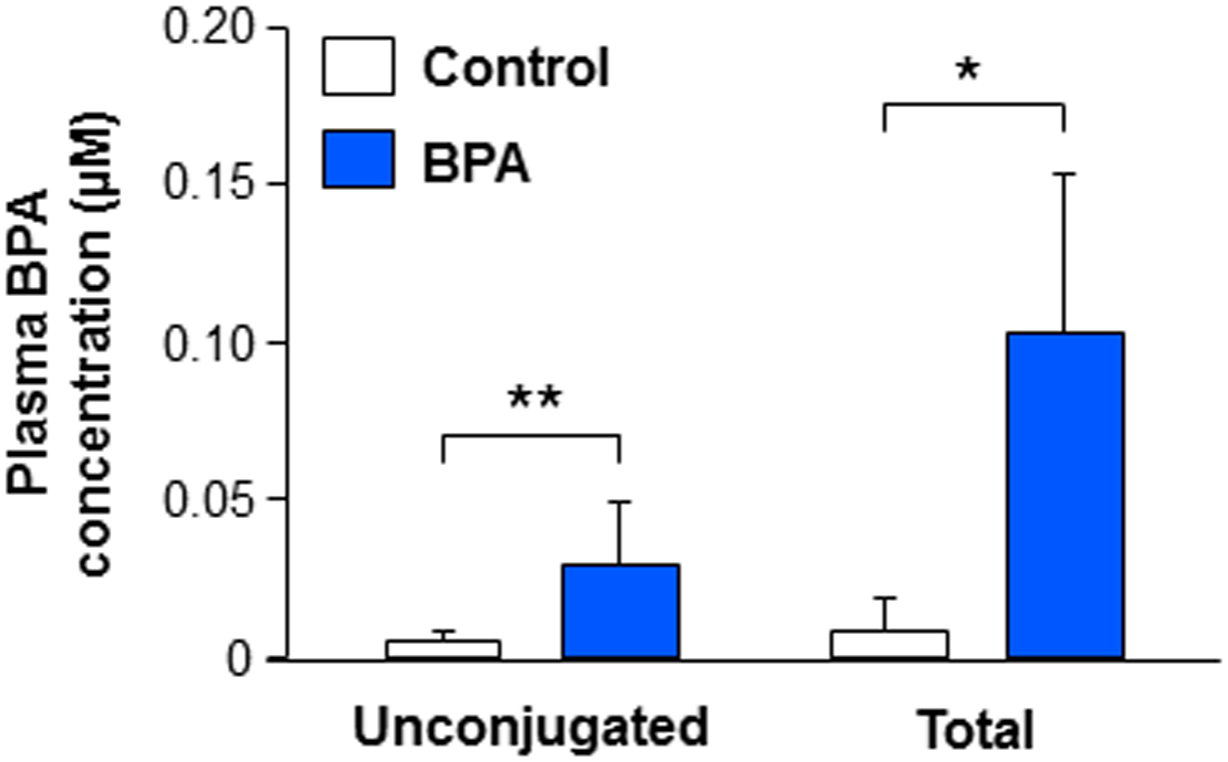
Effect of BPA exposure on plasma BPA concentration in xenografted mice. Plasma levels of BPA were quantified by gas chromatography coupled to tandem mass spectrometry (GC-MS/MS) from castrated Nude male mice xenografted with first trimester human fetal testis (9.1–11.3 GW, mean 10.2 ± 0.2 GW; n = 6–7) and exposed to vehicle (Control) or BPA (10 μM in the drinking water) for five weeks. For each fetus, all the pieces from one testis were grafted in a control mouse and all the pieces from the contralateral testis were grafted in a BPA-treated mouse. Statistical analysis was performed using the Mann-Whitney test. *p<0.05, **p<0.01.

Plasma of BPA-treated mice contained 0.10 ± 0.04 μM total BPA, one third of which was unconjugated (0.038 ± 0.010 μM) (Fig 2). Control mice exhibited detectable concentrations of total and unconjugated BPA (0.02 ± 0.01 μM and 0.007 ± 0.001 μM respectively). In the literature, generally, internal level of BPA is not assayed in both control and treated animals. However, a recent study performed by the National Program of Toxicology carefully evaluated total and unconjugated levels of BPA in the plasma of control rats and reported values in the same order of magnitude as the present results [53]. Importantly, in the present study, both unconjugated and total plasma BPA concentrations were significantly higher in the treated group compared with controls.

#### Histological analyses

After dehydration the tissue pieces were embedded in paraffin wax and sectioned at 5 μm thickness. Serial testes sections were deparaffinized, rehydrated and haematoxylin-eosin-saffron staining or immunohistochemistry were performed as previously described [54,55].

To evaluate the percentage of apoptotic and proliferative germ cells, immunohistochemistry was performed using anti-cleaved caspase-3 (CC3; 1:100, Cell Signaling, 9661S) and anti-Ki67 (1:50, BD Pharmingen, cat 556609) antibodies respectively. Anti-AMH antibody (1:200, Santa Cruz, sc6886) [48] was used to stain the Sertoli cells in order to aid identification of the AMH-negative GC within the seminiferous cords. Immunohistochemistry was conducted using anti-AP-2γ (1:100, Santa Cruz, sc12762) or anti-MAGE-A4 (1:1000, GeneTex, GTX116357) antibodies to determine germ cell differentiation status. The primary antibodies were detected using species-specific secondary antibodies conjugated with a peroxidase activity polymer (ImmPRESS reagent kit; Vector Laboratories, Burlingame, CA). Peroxidase activity was visualized in brown using DAB (3,3’-diaminobenzidine) as substrate or visualized in green using Permanent HRP Green Kit (Zytomed Systems).

To quantify germ cell density, germ cells were identified based on morphological appearance after haematoxylin-eosin-saffron staining and counted in several randomly chosen areas each one measuring 0.464 mm^2^.

All counts were carried out blind using the Histolab analysis software (Microvision Instruments, Evry, France). A minimum of 500 germ cells were counted per testis.

#### Testosterone radioimmunoassay

Serum testosterone concentration of Nude host mice was measured as previously described [56]. The limit of detection was 80 pg/mL. The intra-assay coefficient of variation (CoV) is 2% and the inter-assay CoV is 5%.

#### RNA extraction, reverse transcription and real-time Polymerase Chain Reaction

A piece of each grafted testis was frozen in RLT buffer (Qiagen, Courtaboeuf, France) and stored at– 20°C. Total RNA from frozen samples was extracted using the RNeasy Mini-Kit (Qiagen, Courtaboeuf, France) and reverse transcribed using the High Capacity cDNA Reverse, followed by real-time PCR as previously described (48). The StepOne real-time PCR system (ThermoFisher Scientific) and Quantifast Sybr green (Qiagen) were used for RT–qPCR. The comparative ΔΔcycle threshold method was used to determine the relative quantities of specific key steroidogenic mRNA (*STAR*, *CYP11A1*, *CYP17A1 and CYP19*) using *β-ACTIN*, *RPL0* and *Cyclophilin A (CYPA)* as reference genes. Each sample was run in duplicate. The sequences of oligonucleotides used with SYBR-green detection were designed with Primer express Software (Table 1).

**Table 1:** Sequences of the primers.

Genes	Forward sequence	Reverse sequence
*STAR*	CGTGGTACTCAGCATCGA	TGGGCACAGTTGGGAACA
*CYP11A1*	TGGGTCGCCTATCACCAGTAT	CCACCGGTCTTTCTTCCA
*CYP17A1*	CCACCTTTGCCCTGTTCAAG	GCCAGCATATCACACAATGTACT
*CYP19*	TCACTGGCCTTTTTTCTCTTGGT	GGGTCCAATTCCCATGCA
*β-ACTIN*	TGACCCAGATCATGTTTGAGA	TACGGCGAGAGGCGTACAGG
*RPLP0*	TCCCACTTGCTGAAAAGGTCA	CAAAGGCAGATGGATCAGCC
*CYPA*	CGCGTCTCCTTTGAGCTGTT	ATTTTCTGCTGTCTTTGGGACC

#### Statistical analysis

All data are presented as means ± SEM. The statistical significance of the difference between control and BPA-treated data were evaluated using the non-parametric test: Wilcoxon for paired comparisons and Mann-Whitney for unpaired ones. Statistical significance was set as p<0.05.

### Second trimester human fetal testis xenograft studies

#### Collection of second trimester human fetal testis

Human testes (GW 14–18) were collected from second-trimester fetuses (n = 4), obtained after the medical termination of pregnancy following written informed consent. Gestation was determined by ultrasound scan and subsequent direct measurement of foot length. Ethical approval for obtaining the tissue was given by the South East Scotland Research ethics committee (reference number LREC08/S1101/1).

#### Host mice for xenografting

Male CD1 nude mice (aged 4–6 weeks; n = 64; Charles River UK) were housed in individually ventilated cages with access to soy-free diet and water ad libitum. In preparation for xenografting, mice were anesthetised by inhalation of isofluorane and castrated through a scrotal incision. Castration was performed at least 2 weeks prior to xenografting. Following castration, mice received analgesia (Carprofen; Pfizer) in the drinking water for 3 days post-surgery.

#### Xenografting procedure

Human fetal testis xenografts were performed as previously described [41]. Briefly, human fetal testes were cut into approximately 1 mm^3^ pieces and grafted subcutaneously on either side of the midline of nude mice. Testis tissue pieces from each human fetus testis were grafted into 1–3 host mice. Specific approval for the animal studies, including ethical approval was given by the UK Home office and all procedures were conducted in accordance with the Animal (Scientific Procedures) Act 1986. Mice received corn oil containing either vehicle (absolute ethanol) or BPA (Sigma-Aldrich) by daily gavage at a dose of 0.5 or 50μg/kg of body weight for a period of 5 weeks. Mice were euthanized by inhalation of CO_2_ and cervical dislocation. Blood was extracted by cardiac puncture for testosterone measurements and seminal vesicles were removed and weighed.

#### Testosterone assay

Serum testosterone concentration of Nude host mice was measured as described above for the first trimester. To perform relevant comparisons between values observed in first and the second trimester xenografted mice hosts, all the plasma testosterone extraction and assay were performed by the same investigator.

### Statistical analysis

The method is the same as that used above for the studies of the first trimester testis.

## Results

### Effects of BPA exposure on first trimester human fetal testis

#### Effects of BPA on gonocyte apoptosis and proliferation in first trimester human fetal testis using FeTA model

**Fig 3:**
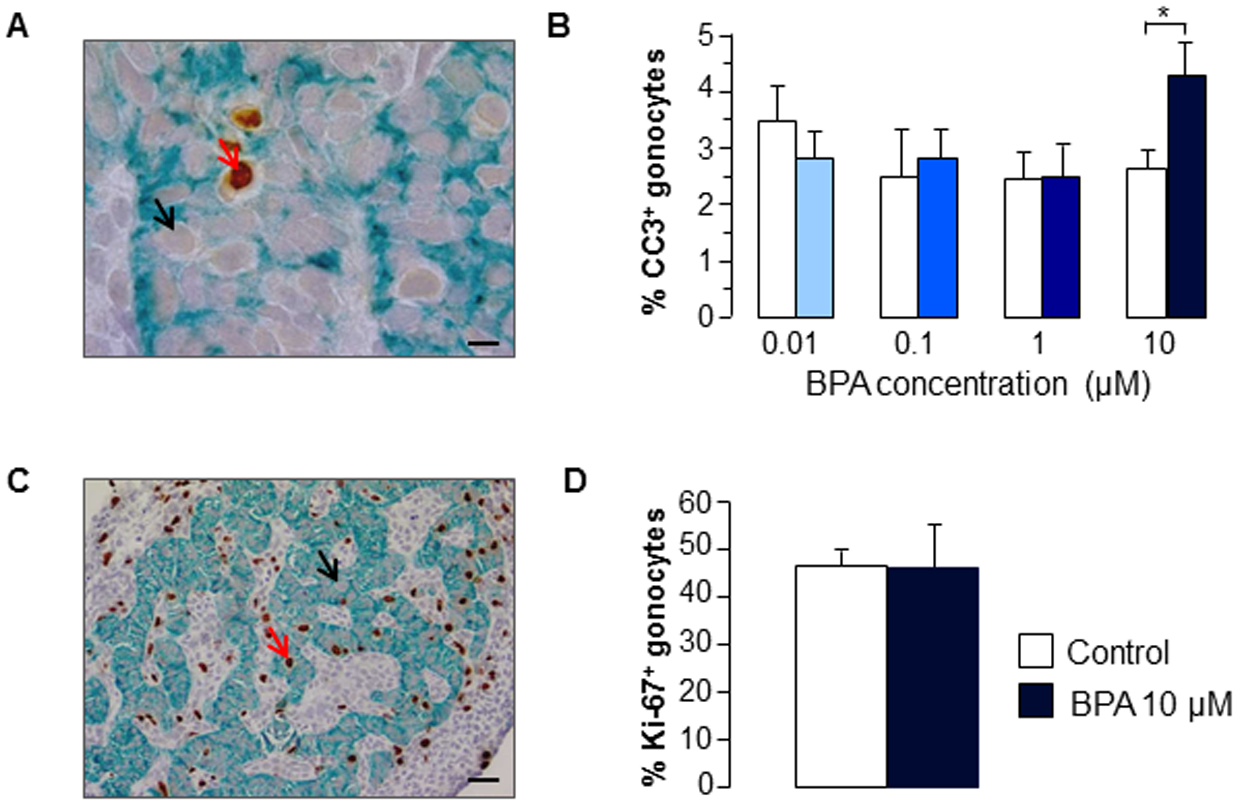
Effect of BPA exposure on germ cell apoptosis and proliferation in first trimester human fetal testes cultured using the FeTA system. Human fetal testes (6–12 GW, mean 8.7 ± 0.6 GW) were cultured using the ex vivo human Fetal Testis Assay system (hFeTA). After 24 hours in control medium, explants were cultured with 100 ng/mL of LH for the 3 subsequent days in the presence of ethanol vehicle (control explants) or BPA at concentrations ranging from 0.01 to 10 μM. Control and BPA-treated explants were paired samples from the same testis. (A) Histological sections after labeling with anti-cleaved caspase-3 antibody (brown) and anti-AMH antibody (green). Positive (red arrows) and negative (black arrows) germ cells can be identified. Scale bar: 10 μm. (B) Quantification of cleaved caspase-3 positive cells (mean ± SEM; n = 4–8). (C) Histological sections after labeling with anti-Ki-67 antibody (brown) and anti-AMH antibody (green). Positive (red arrows) and negative (black arrows) germ cells can be identified. Scale bar: 50 μm. (D) Quantification of Ki67 positive gonocytes (mean ± SEM; n = 4–8).

Human fetal testes from 6 to 12 GW, were cultured for 72 hours in the presence or absence of BPA at various concentrations ranging from 0.01μM to 10 μM to evaluate the effect of BPA on germ cells apoptosis (Fig 3A and 3B). Exposure to 10 μM BPA significantly increased germ cells apoptosis *ex vivo* by 76 ± 37% (n = 8; p<0.05). However, lower concentrations of BPA did not affect germ cells apoptosis.

Moreover, exposure to 10 μM BPA did not affect proliferation of human fetal germ cells based on quantification of immunostaining for Ki-67 (Fig 3C and 3D).

#### Effect of BPA on germ cell apoptosis, proliferation and density in first trimester human fetal testis xenografts

**Fig 4:**
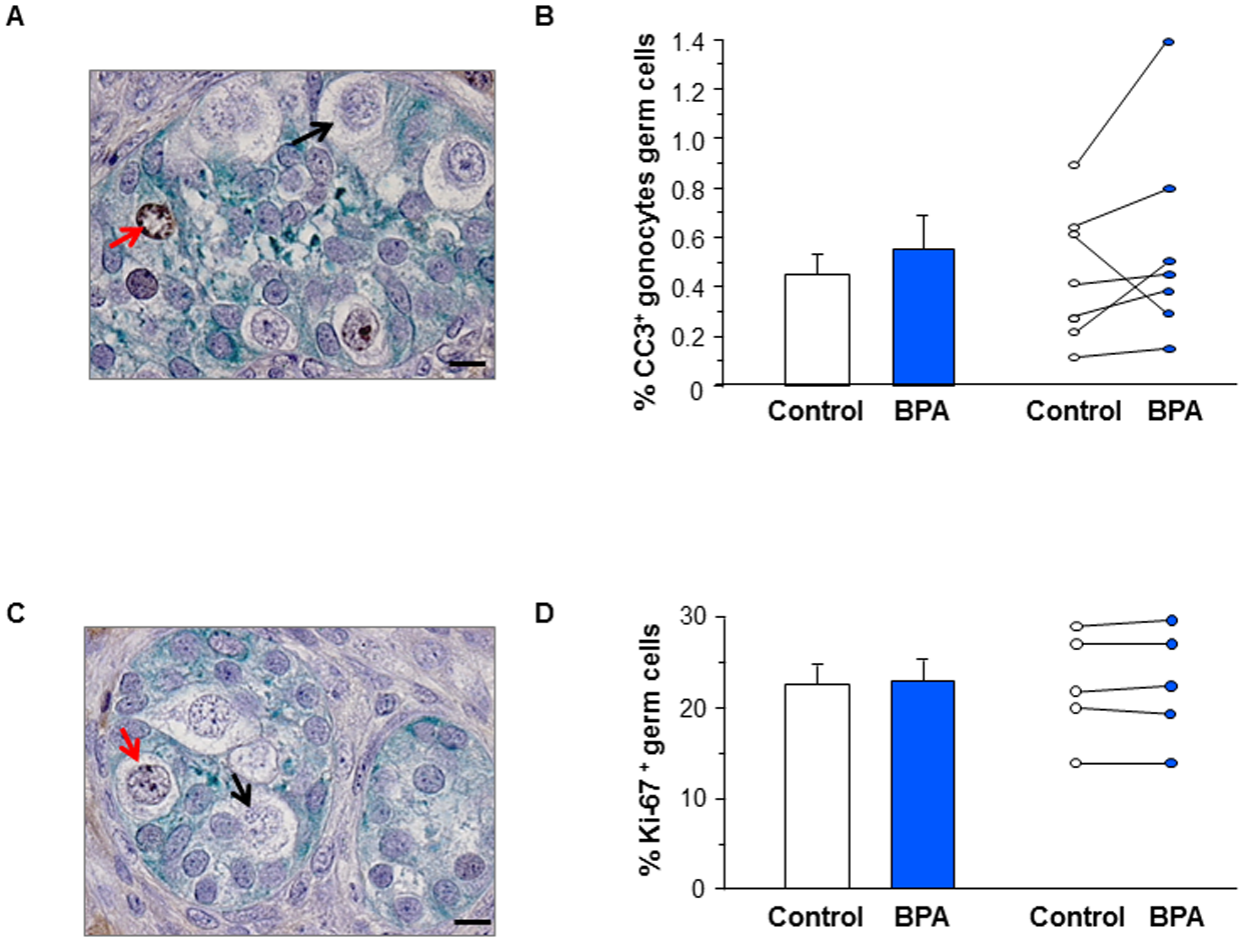
Effect of BPA exposure on germ cell apoptosis and proliferation in first trimester human fetal testis xenografts. Human fetal testes (9.1–11.3 GW) were xenografted into castrate Nude (host) mice. Host mice received vehicle (Control) or 10μM BPA in the drinking water for five weeks. (A) Histological sections of the testis after labeling with anti-CC3 antibody (brown) and anti-AMH antibody (green). (B) Quantification of CC3^+^ cells displayed as mean ± SEM (n = 7) on the left panel and as individual values with a line drawn between the control and the corresponding BPA-treated testis from the same fetus on the right panel. (C) Histological sections after labeling with anti-Ki-67 antibody (brown) and anti-AMH antibody (green). Positive (red arrows) and negative (black arrows) germ cells can be identified. (D) Quantification of Ki67^+^ gonocytes displayed as mean ± SEM (n = 5) on the left panel and as individual values with a line drawn between the control and the corresponding BPA-treated testis from the same fetus on the right panel. Scale bars: 15 μm. Data analyzed using Wilcoxon paired test. For both apoptosis and proliferation, the differences between vehicle and BPA-exposed groups were not statistically significant.

The percentage of apoptotic germ cells (CC3^+^) was increased by 35±22% (n = 7) in the BPA-treated group compared with the controls, although this did not reach statistically significance (Fig 4A and 4B). BPA exposure did not affect the percentage of gonocyte proliferating (Ki67^+^) gonocytes (Fig 4C and 4D).

**Fig 5:**
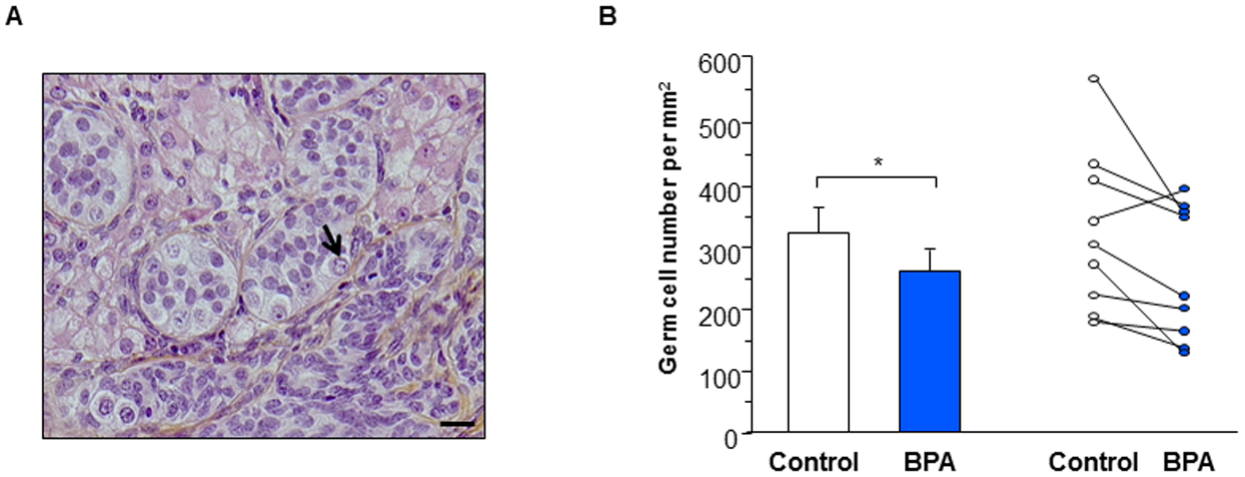
Effect of BPA exposure on germ cell density in first trimester human fetal testis xenografts. Human fetal testes (9.1–11.3 GW) were xenografted into castrate Nude (host) mice. Host mice received vehicle (Control) or 10μM BPA in the drinking water for five weeks. (A) Histological sections after haematoxylin-eosin-saffron staining. Germ cells (black arrow) can be easily identified. Scale bar: 20 μm. (B) Quantification of germ cell density displayed as mean ± SEM (n = 9) on the left panel and as individual values with a line drawn between the control and the corresponding BPA-treated testis from the same fetus on the right panel. Data analyzed using the Wilcoxon paired-test. *p<0.05 compared with control condition.

We hypothesized that a modest but sustained negative effect of BPA on germ cell apoptosis during a longer period would impact on the germ cell density. Indeed, we observed that 5 weeks of treatment with BPA significantly reduced the density of germ cells by 19±7% (n = 9) as compared with control (Fig 5).

#### Effect of BPA on germ cell differentiation in first trimester human fetal testis xenografts

**Fig 6:**
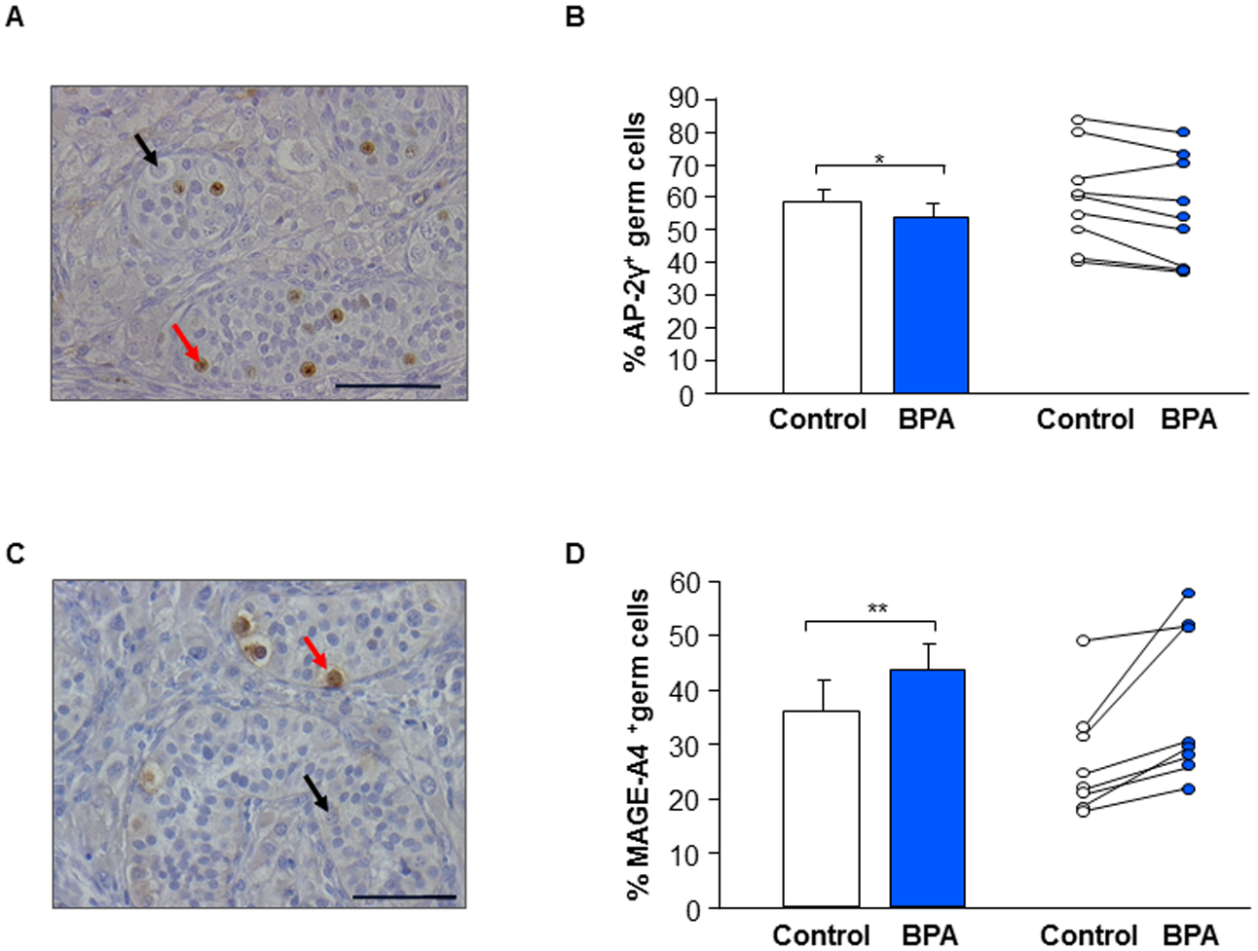
Effect of BPA exposure on germ cell differentiation in first trimester human fetal testis xenografts. Human fetal testes (9.1–11.3 GW) were xenografted into castrate Nude (host) mice. Host mice received vehicle (Control) or 10μM BPA in the drinking water for five weeks. (A) Histological sections of testes after immunostaining for AP-2γ (gonocytes). Positive (red arrows) and negative (black arrows) germ cells can be identified. Scale bar: 60 μm. (B) Quantification of AP-2γ-positive cells displayed as mean ± SEM (n = 9) on the left panel and as individual values with a line drawn between the control and the corresponding BPA-treated testis from the same fetus on the right panel. (C) Histological sections of testes after immunostaining for MAGE-A4 (prespermatogonia). Positive (red arrows) and negative (black arrows) germ cells can be identified. Scale bar: 60 μm. (D) Quantification of MAGE-A4-positive cells displayed as mean ± SEM (n = 8) on the left part and as individual values with a line drawn between the control and the corresponding BPA-treated testis from the same fetus on the right part. Data analyzed using the Wilcoxon paired-test. *p<0.05, **p<0.01.

Using immunohistochemistry, we quantified the percentage of germ cells expressing a gonocyte marker (AP-2γ), and a pre-spermatogonial (MAGE-A4) marker (Fig 6A and 6C). After BPA treatment, percentage of germ cells expressing AP-2γ was significantly decreased by 6.9 ± 2.7% (n = 9) whereas percentage of germ cells expressing MAGE-A4 was significantly increased by 21.2 ± 6.2% (n = 8) compared with control (Fig 6B and 6D).

#### Effect of BPA on hCG-stimulated androgenic production in first trimester human fetal testis xenografts

**Fig 7:**
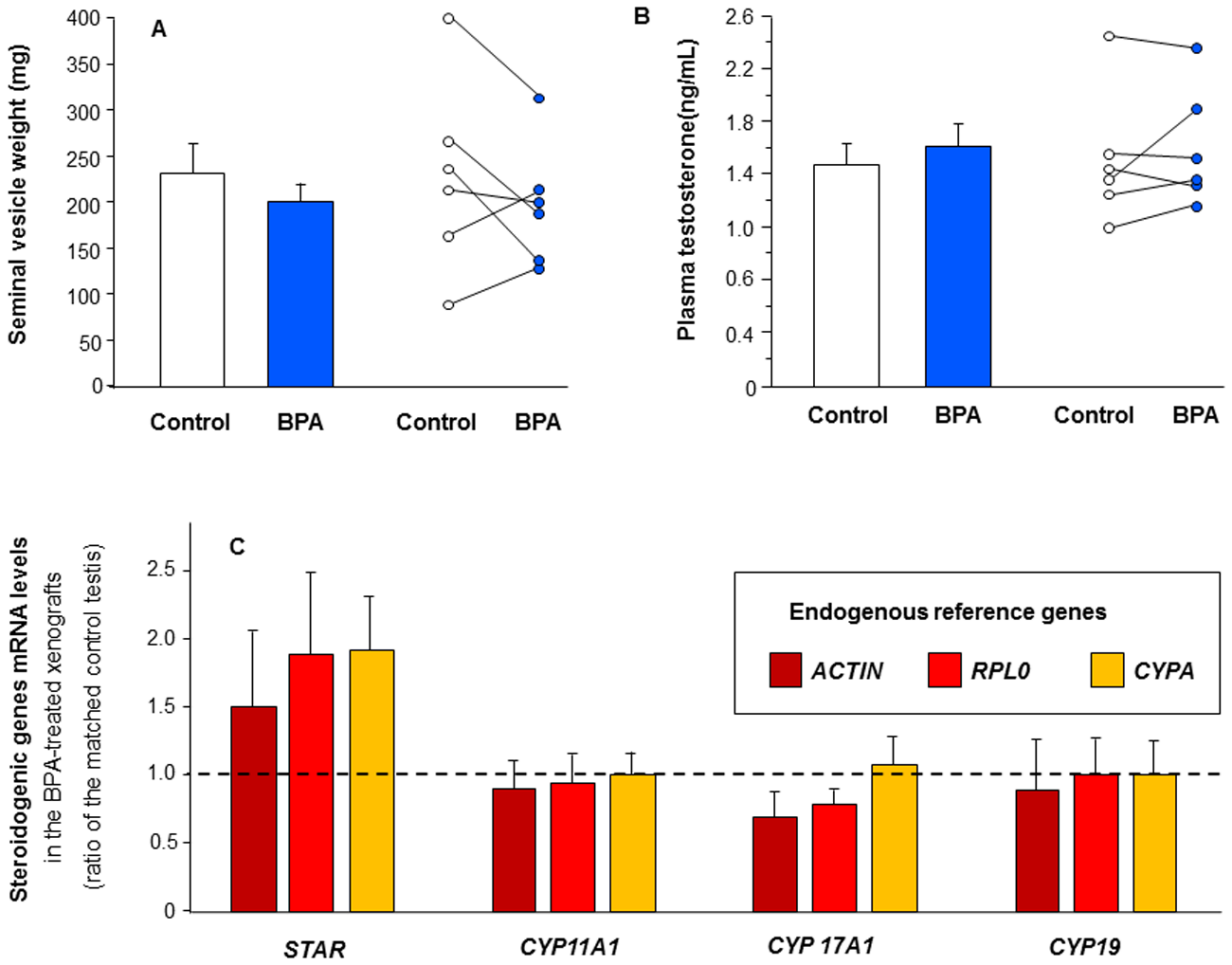
Effect of BPA exposure on plasma testosterone level and seminal vesicle weight in the host mice xenografted with first trimester human testes and on steroidogenic genes expression in the xenografts. Human fetal testes (9.1–11.3 GW) were xenografted into castrate Nude (host) mice. Host mice received vehicle (Control) or 10 μM BPA in the drinking water for five weeks. (A) seminal vesicle weight displayed as mean ± SEM (n = 6) on the left panel and as individual values with a line drawn between the control and the corresponding BPA-exposed testis from the same fetus on the right panel. B) plasma testosterone concentration in host mice displayed as mean ± SEM (n = 6) on the left panel and as individual values with a line drawn between the control and the corresponding BPA-exposed testis from the same fetus on the right panel. C) Expression of key genes in the steroidogenic pathway (STAR, CYP11A1, CYP17A1 and CYP19) using quantitative RT-PCR standardized to either β-ACTIN (ACTIN) or RPLP0 or CYPA as endogenous control. Results are presented as a percentage of the control value (mean ± SEM; n = 4). Data analyzed by Wilcoxon test. No significant difference between BPA-treated and control mice were identified.

We analyzed endocrine function of control xenografts at the end of the experiment. Seminal vesicle (SV) weight (an indirect measure of androgen production) of host control mice was 230 ± 43 mg (Fig 7A) indicating secretion of testosterone during the six weeks of xenografting. This is similar to SV weight (~280 mg) in non-castrated non-xenografted Nude mice whilst castrated non-xenografted Nude mice have SV weights of ~5–10 mg. No significant differences were found in SV weight (Fig 7A) or plasma testosterone level (Fig 7B) between BPA-treated and control mice. It should be noted that SV weight and plasma testosterone concentration were significantly positively correlated both in controls (r = 0.816, n = 6) and in BPA-treated mice (r = 0.845, n = 6).

To confirm the lack of effect of BPA on steroidogenesis, we measured the mRNA levels of various key regulators in the steroidogenic pathway. BPA treatment did not affect the expression of *STAR*, *CYP11A1*, *CYP17A1*, and *CYP19* regardless of the reference gene (Fig 7C).

### Effects of BPA exposure on second trimester human fetal testis

#### Effect of BPA on hCG-stimulated androgenic production in second trimester human fetal testis xenografts

**Fig 8:**
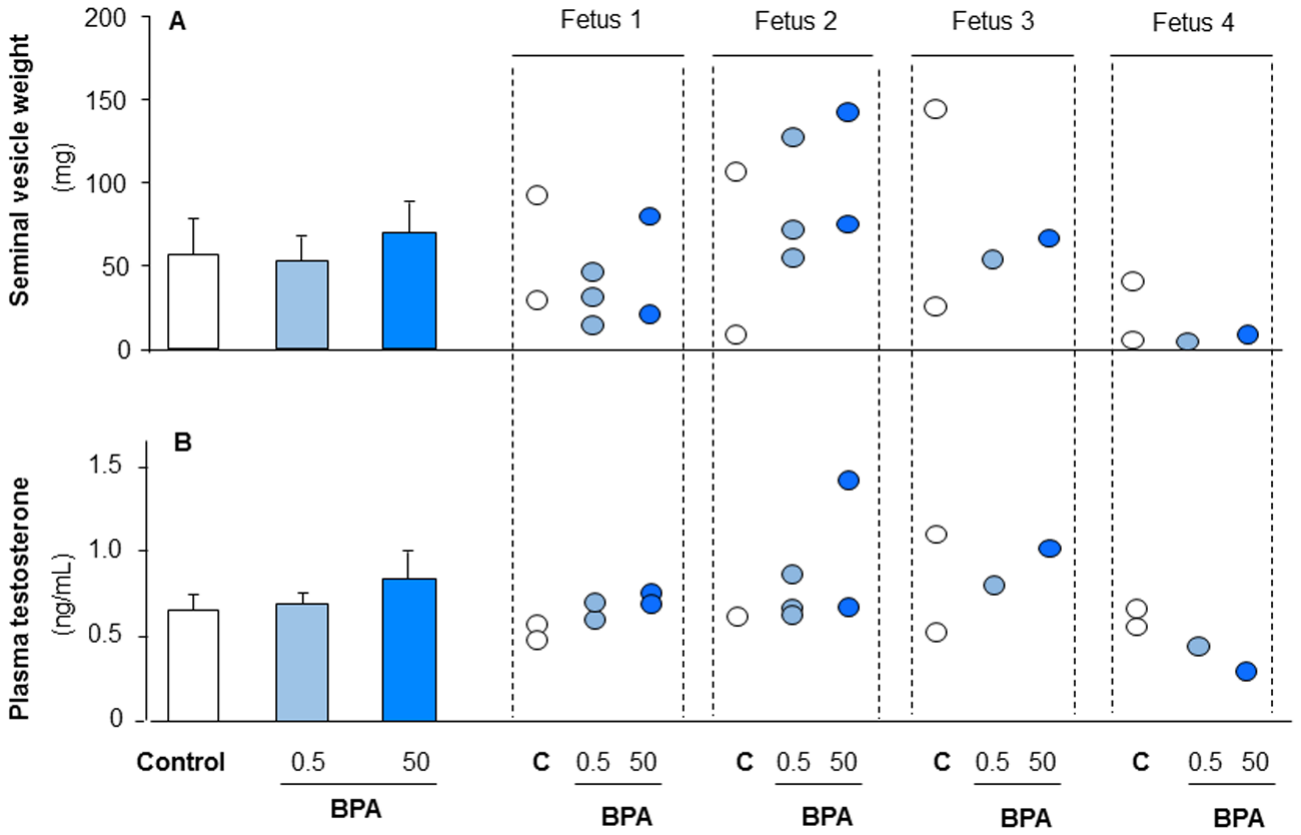
Effect of BPA exposure on testosterone plasma level and seminal vesicle weight in the host mice carrying second trimester human fetal testis xenografts. Human fetal testes (14-18GW) xenografted into castrated Nude (host) mice. Each human fetal testis was grafted into 1–3 host mice which received the same treatment. Host mice received vehicle (Control), BPA (0.5μg/kg or 50μg/kg/d) daily by oral gavage for five weeks. A) seminal vesicle weight as overall mean ± SEM (n = 4) is displayed on the left with individual values on the right. B) plasma testosterone concentration in host mice as overall mean ± SEM (n = 4) is displayed on the left with individual values on the right. Data analysed by Mann-Whitney test. No significant differences were observed for serum testosterone or seminal vesicle weight between BPA-treated mice compared to control.

Seminal vesicle weight and serum testosterone were measured in host mice to determine testosterone production over the grafting period. No difference was observed for seminal vesicle weight (Fig 8A) or serum testosterone (Fig 8B) after 5 weeks exposure to 0.5 or 50 μg/kg/d BPA compared with vehicle-exposed control.

## Discussion

The present study is the first experimental demonstration that exposure to environmentally relevant BPA concentrations induces quantitative and qualitative effects in the development of germ cells in the human fetal testis.

First, using the FeTA system, we demonstrated here that BPA has the potentiality to increase germ cell apoptosis in first trimester human fetal testis *ex vivo* compared with control conditions. However, this was only apparent at higher concentrations (10μM), whilst exposure to lower BPA concentrations (1, 0.1 and 0.01μM) had no effect. Using the FeTA system, we were able to demonstrate a similar effect of BPA exposure on gonocyte apoptosis in the mouse testis (S1 Fig). In this case, a concentration of 0.1 μM BPA was sufficient to significantly increase apoptosis. This demonstrates, for the first time, BPA-induced apoptosis in the fetal germ cells of rodents and human similar to that which has previously in adult rodents. Oral administration of BPA in adult rodent (from 2 μg to 960 mg/ kg/day) induces germ cell apoptosis [57–59] and increases oxidative stress in testis of adult rats at concentrations of 20 and 50 mg/kg/day [16,60]. In the same way a single injection of 50mg/kg BPA induced germ cell apoptosis in the pubertal rat testis via an activation of p38 MAPK, a well-known marker of cellular stress [61]. Furthermore, DNA damage has been demonstrated in cultured CHO-K1 and Chicken DT40 cells exposed to BPA at concentrations ranging from 30 to 600 μM [62,63] and in meiotic germ cells of *C*. *elegans* exposed to 1mM BPA and of mice exposed orally with 10mg/kg/day [64,65]. These cytotoxic effects of BPA may be responsible for the increase in apoptosis and further investigation is required to determine whether such effects on oxidative stress and DNA damages also occurs in male human fetal germ cells. Whilst these results show consistent effects, the concentration of BPA to which the cells/tissues/animals were exposed is often higher than those reported in the serum of humans. To focus on environmental BPA concentrations, we utilized a xenograft model which allows longer term exposure using low doses.

One important question when assessing one ED risk for human health is to appreciate whether the chosen protocol reproduces BPA concentrations relevant to environmental exposure in human. No data are available regarding the BPA level in the plasma of human fetuses during intrauterine life. In the adult, although there remains some uncertainty and dispute, about the level of effective exposure in humans [66,67], the overall consensus is that internal exposure to unconjugated BPA, which is the only biologically active form of BPA [68], is in the range of 0.002–0.043μM (*i*.*e*. 0.5–10μg/L) in blood and serum samples from healthy men and non-pregnant women [69]. Internal BPA concentrations in pregnant women may be slightly higher since different studies reported a mean value of 0.017 μM (3.88μg/L) [69], which this was confirmed by a recent study [70]. Concentrations of unconjugated BPA in umbilical cord blood of newborn are reported to range from 0.0006 to 0.021μM (i.e. 0.14 to 4.76μg/L) with a mean value equal to 0.005 μM (1.12 μg/L) [71]. Lastly, it has been proposed that BPA might accumulate particularly in younger fetuses because of lower metabolic clearance or conjugation at this developmental stage [72]. We took this into consideration when selecting our dosing schedule. In the first series of experiments, it was an administration of BPA via the drinking water and thus, the level of exposure had to be quantified in this model. We observed that the plasma level of BPA, and hence the concentration to which the human testicular xenografts are exposed, was 0.1 and 0.038μM (i.e. 22,8 and 8.7μg/L) for total and unconjugated BPA respectively. In the second series of experiments using host mice carrying second trimester human fetal testis xenografts, we administrated doses of 0.5 and 50μg/kg/day of BPA to the treated host. By comparison, it is interesting to recall that 4 μg/kg/day, is the tolerable dose intake (TDI) estimated by the EFSA. This TDI was derived from a Point of Departure which is the equivalent to a Non Observable Adverse Level Effect (NOAEL) equal to 8,960μg/kg/day *i*.*e*. higher than all the doses we used in the current series of experiments (0.5, 50, 500μg/kg/d). Taken together, these data suggest that the three doses of BPA that we chose are relevant to environmental exposure in human.

With the xenografting model, germ cell density in the grafts from the first trimester was statistically reduced after a 5-weeks exposure to BPA. This suggests that low doses of BPA provoke a weak increase in germ cell apoptosis that is cumulative over the time, ultimately resulting in a decrease in the germ cell number. Whether this degree of germ cell loss is subsequently compensated and the potential clinical significance is uncertain and would require further investigation.

Importantly, we observed that BPA changed not only the germ cell number but also germ cell differentiation status in first trimester testis xenografts. Exposure to BPA significantly decreased the proportion of germ cells expressing AP-2γ a gonocyte marker and pluripotency factor, whilst the proportion of germ cells expressing the pre-spermatogonial marker MAGE-A4 was increased. It is known that the proportion of germ cells expressing AP-2γ decreases during intrauterine life and finally disappears around 36 GW whilst the percentage of germ cells expressing the pre-spermatogonial marker MAGE-A4 increases from the end of the first trimester onwards [37–40,42]. Thus, our results suggest that BPA exposure could accelerate progression of human germ cell differentiation from gonocyte to spermatogonia, although the clinical importance of such a finding is unknown.

This study is the first to investigate the effect of BPA exposure on male germ cells differentiation in human fetal testis; however, there are limited data on BPA effects on male fetal germ cells differentiation in rodents. Using mouse ES cells, Aoki and Takada have shown that BPA at 50 μM induces an up-regulation of *Stra8*, a meiotic entry gene, an increase in ovarian markers expression such as *Wnt4* and *Foxl2*, and a decrease in testicular markers expression such as *Sox9* and *Fgf9* [73]. Another study has described that treatment of pregnant mice with BPA at 40, 80 and 160μg BPA/kg body weight/day may induce epigenetic and gene expression modifications in fetal germ cells in both sexes [74]. This may be related to the fact that BPA alters the meiotic process in fetal oocytes in humans and non-human primates resulting in the production of chromosomally abnormal eggs [75,76].

We also investigated the effect of BPA exposure on androgenic production in the first and the second trimester human fetal testis. Androgen action during fetal life is important for the process of masculinization of the male fetus. It has been shown in rats that a decrease in testosterone synthesis or action during a critical period (15.5 and 18.5 day post conception), named “the masculinizing programming window” (MPW), causes masculinization defects that occur from 18.5 dpc onwards, whilst a decrease in androgen production after this period does not affect the ontogenesis of male reproductive organs but reduces their final size [77,78]. In humans, the putative MPW extends from 8 to 14 GW [77,79] and masculinization begins on GW 10 with subsequent growth throughout intrauterine life.

Importantly, we have demonstrated that five weeks exposure to low BPA concentrations does not alter the hCG-stimulated testosterone secretion by xenografted human fetal testis. This was observed for both first trimester and second trimester xenografts although there were differences in the exposure route, the BPA doses and the grafting procedure between the two series of experiments. Taken together, these results suggest that the effect of BPA on germ cells described in the present study is unlikely to result from altered testosterone production despite the importance of testosterone for gonocyte development [80,81]. The absence of a BPA effect on hCG-stimulated testosterone production in the xenografting model is also in agreement with previous results obtained using the FeTA system for which, we and others have shown that concentrations of BPA lower than 10μM do not alter LH- or hCG-stimulated testosterone production by human fetal testes [15,32]. However, using the same FeTA approach we have also shown that 0.01μM and higher BPA concentrations reduce basal testosterone production of the human fetal testes [31,32]. Given that hCG stimulation more accurately reflects the physiological situation, we would consider the results obtained in the presence of LH or hCG to be more representative of the situation in human pregnancy than those obtained under basal conditions. However, hCG plasma concentrations change during development and can vary considerably from one fetus to another [79] thus making precise replication of these *in vivo* conditions challenging. Another challenge is that the endocrine milieu contains a mixture of LH and hCG with a ratio changing from the end of the first trimester onwards.

Limited epidemiological studies have resulted in conflicting results in relation to associations between BPA exposure *in utero* and male reproductive abnormalities. A Chinese study involving the sons of workers who were occupationally exposed to BPA during pregnancy were shown to have a shorter anogenital distance (an index of fetal testicular testosterone production) compared to a control group [82]. However, no increase in BPA concentration in umbilical cord blood was observed in newborns with cryptorchidism [71], and no association was found between concentrations of BPA and testosterone in umbilical cord blood in a population-based cohort [83].

## Conclusion

This is the first study performed by the same group comparing organ culture and xenograft approaches for determining the effects of environmental chemical exposures in the human fetal testis. Importantly, both *ex vivo* (FeTA) and *in vivo* (xenografts) resulted in similar effects in terms of germ cell loss as a result of BPA exposure, whilst hCG-stimulated testosterone production was unaffected in both systems. They appear as two complementary approaches since FeTA can be used for high-throughput screening, dose-response and mechanism of action studies, whereas xenografting can be used for long-term *in vivo* exposure studies.

Although the fetal testis is well described as a potential target of EDs [15], we developed here, using the xenograft model, the first experimental *in vivo* study of the effects of BPA on the development and function of the human fetal testis using BPA exposures that have been reported to reflect environmental exposure. With these conditions, BPA did not affect hCG-stimulated androgenic function of the xenografted fetal testis but we did identify effects on fetal germ cell number and differentiation that could potentially impact on fertility at adulthood.

## Supporting information

S1 Fig Effect of BPA exposure on germ cell apoptosis in mouse fetal testes cultured using the FeTA system. Testes were removed from NMRI mice at 12.5 day-post conception and cultured as previously described using the FeTA system [29,31,48,49]. After 24 hours in control medium, explants were cultured under basal conditions for the subsequent 24 hours in the presence of ethanol (vehicle control) or 0.1 μM BPA. The effect of BPA on germ cell apoptosis was estimated by the comparison between the percentage of cleaved caspase-3 positive gonocytes in the testis cultured in the presence of BPA and that measured in the other testis from the same fetus cultured without BPA (control) as previously described [29,48]. Quantification of cleaved caspase-3 positive cells are presented as mean ± SEM (n = 9) in the left panel and as individual values with a line drawn between the control and the BPA-exposed testis from the same fetus in the right panel. Data was analysed using the Wilcoxon paired test. The increase in apoptosis in response to 0.1μM BPA was statistically significant (p = 0.027).
(TIF)
