# Supplementary figures and images for: Effects of environmental Bisphenol A exposures on germ cell development and Leydig cell function in the human fetal testis
Source: PLoS One. 2018 Jan 31;13(1):e0191934. doi: 10.1371/journal.pone.0191934 (PMC5791995; doi:10.1371/journal.pone.0191934)

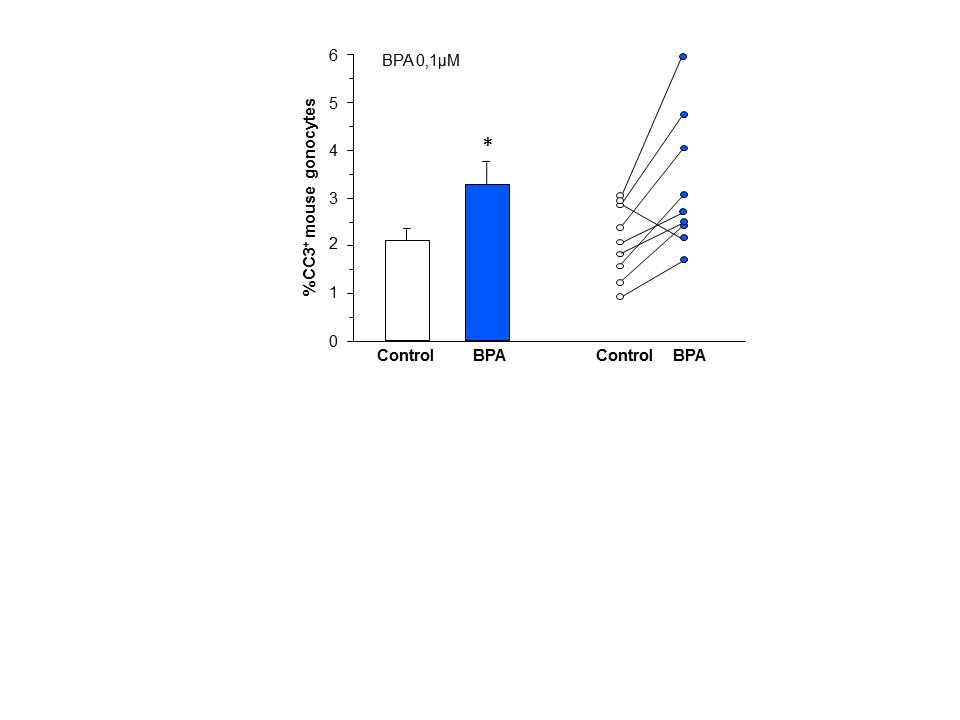

Supplement: S1 Fig — Testes were removed from NMRI mice at 12.5 day-post conception and cultured as previously described using the FeTA system [29,31,48,49]. After 24 hours in control medium, explants were cultured under basal conditions for the subsequent 24 hours in the presence of ethanol (vehicle control) or 0.1 μM BPA. The effect of BPA on germ cell apoptosis was estimated by the comparison between the percentage of cleaved caspase-3 positive gonocytes in the testis cultured in the presence of BPA and that measured in the other testis from the same fetus cultured without BPA (control) as previously described [29,48]. Quantification of cleaved caspase-3 positive cells are presented as mean ± SEM (n = 9) in the left panel and as individual values with a line drawn between the control and the BPA-exposed testis from the same fetus in the right panel. Data was analysed using the Wilcoxon paired test. The increase in apoptosis in response to 0.1μM BPA was statistically significant (p = 0.027). (TIF) [file pone.0191934.s001.TIF]
